# How has formal institutions influenced opportunity and necessity entrepreneurship? The case of brics economies

**DOI:** 10.1016/j.heliyon.2020.e04931

**Published:** 2020-09-14

**Authors:** Thomas Bilaliib Udimal, Mingcan Luo, E Liu, Nicholas Oppong Mensah

**Affiliations:** a School of Economics and Management, Southwest Forestry University, Kunming, Yunnan China 650224; b School of Agriculture Technology, University of Energy and Natural Resources, Sunyani Ghana

**Keywords:** Opportunity entrepreneurship, Necessity entrepreneurship, Female opportunity entrepreneurship, Male opportunity entrepreneurship, Female necessity entrepreneurship, Male necessity entrepreneurship, Economic development, Economic growth, Employment, Inequality, Poverty

## Abstract

Using a panel data of BRICS economies, this study looks at how formal institutions have influenced various types of entrepreneurship. The study concentrated mainly on the following formal institutional factors: fiscal freedom, business freedom, property rights, financial freedom, labour freedom, and investment freedom. For the opportunity entrepreneurship, the result is not statistically significant. With the exception of business freedom, the remaining institutional factors have negative relationship with opportunity entrepreneurship. Fiscal freedom and business freedom show negative significant relationship with necessity entrepreneurship. The result also shows that business freedom has a negative significant effect on female opportunity entrepreneurship. The study further reveals that fiscal freedom, business freedom and financial freedom have a negative significant effect on men necessity entrepreneurship. Population growth has a positive effect on all categories of entrepreneurship whiles unemployment contributes negatively to all categories of entrepreneurship.

## 1 Introduction

The concept of self-ownership in an economic environment is highly dependent on the economic freedom. Self-ownership guarantees the individuals the right to choose- to decide how their time, resources and talents are used to shape their lives. The main elements of economic freedom are personal choice, voluntary exchange, open markets, and clearly defined and enforced property rights. Individuals enjoy economic freedom when they are permitted to choose for themselves and enter into transactions as long as their activities do not harm other persons or properties (Gwartney, 2002). Economic freedom promotes individual choices as individuals will be permitted to decide for themselves rather than having ideas imposed on them by the political process or the use of violence, theft, or fraud by others. However, this is often not case in many economies as institutions that are supposed to promote economic freedom are rather stifling it. It is against this backdrop that the study seeks to look at how formal institutions have influenced entrepreneurial activities in the BRICS (Brazil, Russia, India, China and South Africa) economies.

The study seeks to look at how formal institutions have influenced various kinds of entrepreneurship and across gender groupings in entrepreneurship. Some of the research questions to consider in this study are: (1) how has formal institutions influenced opportunity entrepreneurship. (2) How has formal institutions influenced male and female opportunity entrepreneurships? The study has also included GDP, unemployment and population as economic indicators to measure how they have influenced entrepreneurship.

Entrepreneurship has taken a centre staged in the global economic activities. The global crisis has led to the phenomenon where individuals have decided to take their destinies into their own hands by pursuing opportunities in the global space. As way of lessening the burden on governments, various initiatives are kept in place to ensure that entrepreneurs succeed. Key among the initiatives is putting in place appropriate institutional frameworks to ensuring that entrepreneurs succeed. The argument has always been that entrepreneurial activities promotes economic growth and development (Minniti, 2008), but entrepreneurship can only play a meaningful role in a nation's economic growth and development when institutional policies are formulated to favour its operations. The economic crisis experienced has led to the reformulation of national policies to favour entrepreneurial activities (Bjørnskov and Foss, 2013; Baumol and Strom, 2007; Levie et al., 2014). Institutional theory provides the basis for the role of institutions in entrepreneurship (North, 1994). The theory is based on the assumption that environment does not only influences an individual decision to become an entrepreneur but also the nature of venture one engages in and subsequently influences growth and the development of the country (Minniti and Lévesque, 2008; Baumol, 1996).

There are two types of entrepreneurs, thus opportunity and necessity entrepreneurs (Reynolds et al., 1999). The two are different as circumstances that drive an individual into each of them are completely different. Opportunity entrepreneur is an individual who goes into entrepreneurship as a result of prevailing attractive business opportunities whilst necessity entrepreneurs emerge as a result of absence of opportunities so individuals then take their destinies into their own hands by becoming entrepreneurs. Extant literature have delved into the two types of entrepreneurship by looking at the factors that influence each in the generic analysis of institutional environment (McMullen et al., 2008; Valdez and Richardson, 2013). These studies did not consider the effect of formal institutions on the two types of entrepreneurship at the emerging economies level making it difficult to generalized the findings. The study by Fuentelsaz et al. (2015) looked at the effect of formal institutions on the two types of entrepreneurship globally. The study did not also consider the gender component of each type of entrepreneurship, thus opportunity and necessity entrepreneurs who are females and males. Aside looking at the influence of formal institutions on opportunity and necessity entrepreneurship this study extends the discussion by looking at how the same institutions influence males and female in becoming opportunity and necessity entrepreneurs. This approach has become necessary because of unequal access by males and females to state institutions in most countries. Putting the two sexes together for an analysis does not provide specific direction for policy hence this approach. This study only considers BRICS nations as they have almost the same growth characteristics and putting them together for an analysis will provide a true picture of influence of formal institutions on their entrepreneurial landscape unlike previous studies where such distinction was not made. In addition, the study has included economic indicators such as GDP (Gross Domestic Product), unemployment, and population growth to measure how they have influenced entrepreneurship the approach that has not been explored in extant literature. According to (Acs, 2006; Acs et al., 2008), opportunity entrepreneurship has a positive significant effect on a country's per capita GDP and improves the innovative capacity of the country whereas necessity entrepreneurship affects them negatively (Wennekers et al., 2005). However, the opposite effect has not been considered hence its inclusion. Furthermore, the disparities in opportunities with respect to gender warrants the segregation analysis to inform policy where much efforts should be directed in order to make a desire impact. Already, there are calls for policies to be tailored towards the development of opportunity entrepreneurship as it is seen to contribute more to economic growth and development compared to necessity entrepreneurs but the gender component of it has not received attention (Acs, 2006; Acs et al., 2008). They argued that individuals who become opportunity entrepreneurs consider many factors, which include the institutional environment of their operation.

The main contributions of this study are in several folds. First, we contribute to discussions on entrepreneurship development by looking at it from the perspective BRICS economies, as they have almost similar growth characteristics as emerging economies. We add to the discussion by including more institutional elements and economic indicators, which were absent in previous studies. We add to the debate by looking at gender segregation of each of the types of entrepreneurship. We argue that the influence of formal institutions vary across types of entrepreneurship and across gender hence the segregation. The basis for this approach is from extant literature, which argued that there are gender disparities in entrepreneurship process especially in accessing capital, enterprise growth orientation among others (Guzman and Kacperczyk, 2019; Canning et al., 2012). This approach will afford policy makers the opportunity to formulate more focused policies in addressing the institutional challenges confronting the development of entrepreneurship.

## 2 Literature review

Literature on entrepreneurship is so diverse but they all find their convergence on the work of Schumpeter (1934) where entrepreneurs are seen as innovators or imitators. There are different classifications of entrepreneurship: productive and non-productive; entrepreneurs with growth mindset and those without; entrepreneurship aimed at high-growth activities; formal and informal entrepreneurship (Baumol, 1996; Baumol and Strom, 2007; Dau and Cuervo-Cazurra, 2014). One classification featured in the seminal works of (Shane et al., 1991; Reynolds and Miller, 1992; Reynolds et al., 2003) where they looked at entrepreneurship from opportunity and necessity perspective. In opportunity entrepreneurship, the entrepreneur identifies opportunities in the environment and evaluates them; based on their merits he or she then commits resources into the venture. Necessity entrepreneurship is situation where an individual becomes an entrepreneur due to lack of opportunities. In terms of their effect on economic development and growth, opportunity entrepreneurship has a great effect compared to necessity entrepreneurship. According to Reynolds et al. (2003) opportunity entrepreneurship and necessity entrepreneurship affect the following sectors of the economy differently: (1) job creation; (2) out-of-country export expectations; (3) intention to replicate existing business activities versus the creation of a new niche; (4) market share in different business sectors. The study by Acs and Varga (2005) showed that opportunity entrepreneurship has a positive significant effect on economic growth and development. They however noted that necessity entrepreneurship does not promote economic development. According to McMullen et al. (2008), different institutions have different effects on opportunity and necessity entrepreneurships. Opportunity entrepreneurship has a positive significant effect on a country's per capita GDP and improves the innovative capacity of the country whereas necessity entrepreneurship affects them negatively (Wennekers et al., 2005). In the countries where much emphasis is placed on opportunity entrepreneurship, growth is recorded in per capita GDP and other growth indictors, compared to necessity entrepreneurship (Acs, 2006; Acs et al., 2008). The studies have concluded that when a large proportion of population engages in opportunity entrepreneurship relative to necessity entrepreneurship, economic development and growth are expected. In effect, countries where opportunity entrepreneurship is pursued are expected to experience relative development. According to Fuentelsaz et al. (2015) formal institutions affect opportunity and necessity entrepreneurship differently and therefore recommended for policies to be directed to the opportunity entrepreneurship as it has the greatest impact on economic growth.

As much as above studies are important in understanding the effect of opportunity entrepreneurship and necessity entrepreneurship, none has looked at the gender dimension of opportunity entrepreneurship and necessity entrepreneurship. This study extends the previous studies by looking at the gender dimension and the inclusion of economic indicators.

### 2.1 Institutions

Several factors come into play when entrepreneurship is tabled for discussion. They include perspectives from the economic, psychological, organizational and sociological or institutional (Verheul et al., 2002; Vergés, 1999). The sociological or institutional perspective has argued that the interest to start a business originates from the socio-cultural environment of an individual (Bruton et al., 2010). Studies by (North, 1994; Fritsch and Storey, 2014; Dau and Cuervo-Cazurra, 2014; Urbano and Alvarez, 2014) looked at differences in entrepreneurship activities between countries in relations to their institutions and concluded that differences were because of differences in institutions.

Institutions shape the political and socio-economic environment by ensuring that structures kept in place are working. Good institutions encourage investments. Good institutions eliminate opportunistic behaviour as offenders are not left unpunished. Good institutions build individuals' confidence in the exchange environment as risk are reduced to the barest minimum (North, 1994). It is argued that the degree and quality of entrepreneurship in a country is depended on its institutions (Bruton et al., 2010; Sobel, 2008). Good institutions give the entrepreneurs and the will-be entrepreneurs confidence to pursue emerging opportunities knowing there are institutions to guide against opportunistic behaviours. Institutions that are more binding are the formal institutions. They are political, economic and legal rules formulated to put restrictions on individuals' behaviour to facilitate exchanges. They are more nationalistic in scope. Informal institutions though binding to certain extent there are however, enforcement challenges as it is based on the beliefs, value system, and behaviour of individuals (Sobel, 2008).

Due to the complex nature of informal institutions, as it varies from society to society and country to country in this study, we limited ourselves to formal institutions, as their application is quite general across countries and regions.

The study by Gnyawali and Fogel (1994) identified a host of environment factors, which are key in entrepreneurship pursuit. They include: (1) entrepreneurial and business skills; (2) socioeconomic conditions; (3) financial and non-financial assistance; (4) government policies and procedures. The essence of government policies is to ensure that business environment is freed of any bottlenecks that have a likelihood of frustrating entrepreneurs. It reduces the risk in business environment so that entrepreneurs can be guaranteed of minimum risk.

In this study the following institutional factors considered for analysis are: (1) business freedom; (2) labour freedom; (3) fiscal freedom; (4) property rights; (5) investment freedom; and (6) financial freedom. We therefore argue that formal institutions devoid of manipulations by the state would encourage opportunity entrepreneurship but would not be of much importance in necessity entrepreneurship.

## 3 Hypotheses development

### 3.1 Property rights

It measures the degree to which an individual has the right to own a private property, protected by well-defined laws that can fully be enforced. It shows the level at which a country's laws guarantee private property ownership. The ability of individuals to enforce contracts, the independence of the judiciary and the level of corruption within the judiciary are key in ensuring property rights. The more individuals are sure of protection of their properties by the laws of the country, the higher the score for “property rights”) and vice versa (Unit, 2008). Property rights guarantee entrepreneurs of their investment (Baumol, 1996). According to (Bjørnskov and Foss, 2013) (North, 1994) a well-established legal system and a fair judiciary system devoid of partiality are essential in the growth of economy as it encourages investment. A more secured legal regime will be a source of encouragement for people to engage in entrepreneurship. Both opportunity and necessity entrepreneurship will thrive better in an environment that gives protection to private property ownership. By looking at entrepreneurship from the opportunity and necessity, opportunity entrepreneurs stand the risk of losing much in the environment with weak property rights protection as their aspirations are for growth and employment generation, which the commit much capital (Reynolds et al., 2003; Hessels et al., 2008; Estrin et al., 2013; Levie and Autio, 2011). Also for women entrepreneurs, private property rights will promote female opportunity entrepreneurship, as they will be guaranteed of safety of their investment. We argue that laws guaranteeing private property ownership will promote opportunity entrepreneurship as that will create an enabling environment for their activities. Better property rights protections and contract enforcement for ordinary citizens and broad access to economic opportunities will spur private investments (P.Todaro and Smith, 2015). Compare to necessity entrepreneurs who only resort to entrepreneurship as a last resort, they invest substantially in capital items so they prone to much risk in an environment with weak property rights protection (Levie and Autio, 2011).

Based on the above exposition we therefore hypothesize that:

H1a *Higher private property protection has a positive significant effect on opportunity entrepreneurship*

H1b *Higher private property protection has a positive significant effect on female opportunity entrepreneurship*

H1c *Higher private property protection has no statistical significance on the necessity entrepreneurship*

H1d *Higher property protection has a positive significant effect on the relative presence of female opportunity entrepreneurship compared with male opportunity entrepreneurship.*

### 3.2 Fiscal freedom

It measures the tax burden on individuals and entities imposed by the central government. It has the following components: the top tax on individuals' income; top tax rate on corporate income; total tax burden as a percentage of a country's GDP (Unit, 2008). According to Dean and McMullen (2007) over taxation squeezes money from entrepreneurs, which ends up becoming disincentive. According to (McMullen et al., 2008; Bowen and De Clercq, 2008), tax affects entrepreneurship behaviour negatively. Tax burden siphons working capital from entrepreneurs making them unable to engage in expansion (Estrin et al., 2013). Based on this assertion we therefore argue that fiscal freedom will lead to growth in opportunity entrepreneurship.

But, according to Hessels et al. (2008) the activities of necessity entrepreneurship are more imitative in nature. As a result they necessity entrepreneurs will benefit less from income meant for innovative activities (McMullen et al., 2008; Schumpeter, 1934). Based on the above assertion, we argue that ensuring fiscal freedom in an economy would boost the activities of opportunity entrepreneurs but will be of less significance to necessity entrepreneurship.

We therefore hypothesize that:

H2a *Higher fiscal freedom has a positive significant effect on opportunity entrepreneurship*

H2b *Higher fiscal freedom has no a statistical significance on necessity entrepreneurship*

H2c *Higher fiscal freedom has a positive significant effect on female opportunity entrepreneurship*

H2d *Higher fiscal freedom has a positive significant effect on the relative presence female opportunity entrepreneurship compared with male opportunity entrepreneurship.*

### 3.3 Business freedom

It measures the ability of an individual to start, operate and close a business of their choice. The following is the criteria on which it is measured: starting a business, thus procedures (number); starting a business, thus time (days); starting a business, thus cost (% of income per capita); starting a business, thus minimum capital (% of income per capita). The rest are: obtaining a license, thus procedures (number); obtaining a license, thus time (days); obtaining a license, thus cost (% of income per capita); closing a business, thus time (years); closing a business, thus cost (% of estate); and closing a business, thus recovery rate (cents on the dollar) (Unit, 2008).

The freedom in business environment promotes entrepreneurial activities as it reduces the stress one goes through in engaging in a business activity (Publishing, 2002; Heckelman, 2000). Rigid business environment is reported to have a detrimental effect on the success of businesses (Spencer and Gómez, 2004; Levie and Autio, 2011). Cumbersome business procedures are enough to discourage one from becoming an entrepreneur (Grilo and Thurik, 2005). According to Klapper et al. (2006) complex administrative procedures are impediment to the creation of businesses.

From the perspective of signaling theory Waldman (2016), we looked at the effect of business freedom on each type of entrepreneurship and also from the gender perspective. From the signaling theory, we argue that cumbersome administrative procedures is a source of discouragement to entrepreneurs (Levie and Autio, 2011). However, this signal will only play a significant role in the activities of opportunity entrepreneurship and less in the necessity entrepreneurship as the latter only goes into entrepreneurship as means of survival and not necessarily with growth mindset and are least concerned about procedures or penalties (Brixy et al., 2009). A study by Amin and Haidar (2012) in support of the above argument noted that cumbersome business procedures rather promotes informal sector as they often do not register their operations as way of avoiding complex procedures. Dau and Cuervo-Cazurra (2014) however argued that entrepreneurs with growth mindset are not able to avoid the registration process no matter how cumbersome the procedures are as their size will make them visible to government agencies. At certain stage of business growth owners take personal initiatives to register their operations to enable them enjoy limited liability, which non-registered entities do not enjoy (Levie and Autio, 2011). Having a mindset of growth compels entrepreneurs to comply with business regulations. Since opportunity entrepreneurs have a growth mindset, they stand to benefit more from a greater business freedom compared to necessity who are into entrepreneurship just for survival (Reynolds et al., 2003). We therefore, argue that simplified administrative procedures will boost opportunity entrepreneurship but will be of less significance to the necessity entrepreneurship.

Based on the above exposition, we therefore hypothesize that:

H3a *Higher business freedom has a positive significant effect on opportunity entrepreneurship*

H3b *Higher business freedom has a positive significant effect on female opportunity entrepreneurship*

H3c *Higher business freedom has no statistical significance on the necessity entrepreneurship*

H3d *Higher business freedom has a positive significant effect on the relative presence female opportunity entrepreneurship compared with male opportunity entrepreneurship.*

### 3.4 Labour freedom

The labour freedom looks at the legal and regulations guiding the labour market. It has the following components: ratio of minimum wage to the average value added per worker; hindrance to hiring additional workers; rigidity of hours; difficulty of firing redundant employees; legally mandated notice period, and mandatory severance pay. There are established regulations to guide the employees and employers. This is to ensure that salary, working conditions and mode of compensations are well spelt out. Rigidity in the labour market limits the ability of entrepreneurs to negotiate the working conditions with prospective employees as a result they will not commit resources to the venture (McMullen et al., 2008). Entrepreneurs attach so much importance to controlling of the activities so anything that will limit their ability to control will serve as an impediment to their success (barrier to entry) (Mueller and Thomas, 2001; Asongu, 2014). A regulated labour market will restrict the movement of labour so opportunity entrepreneurs will be demotivated to pursue their dreams as they can hardly leave their jobs due to opportunity costs (Levie and Autio, 2011; McMullen et al., 2008). Restriction in labour market stifles opportunity entrepreneurship. Necessity entrepreneurship is however not affected by labour market restrictions, as there are already limited alternatives. With the mindset of growing their business, opportunity entrepreneurs may in future engage more labourers compared to necessity entrepreneurs (Reynolds et al., 2003). A more flexible labour market will be in the interest of opportunity entrepreneur as they have growth aspirations that will require more labourers (Román et al., 2013). Issues about the labour market will be of concern to entrepreneurs who have plans to engaging labourers and since necessity entrepreneurs only engages in entrepreneurship as a means of survival, labour market will be of little concern to them (Reynolds et al., 2003). According to Román et al. (2013) labour market rigidity turns to promote the activities of smaller businesses as a means to avoid high hiring cost. Given the nature of necessity entrepreneurship and the mindset behind its formation a rigid labour market will encourage its activities (Reynolds et al., 2005). A more flexible labour market will lessen the activities of necessity entrepreneurs as a range opportunities will be available to keep individuals who would have gone into necessity entrepreneurship. We therefore contend that higher labour freedom would favour opportunity entrepreneurship. Based on this assumption, we therefore, formulated the following hypotheses:

H4a *Higher labour freedom has a positive significant effect on opportunity entrepreneurship*

H4b *Higher labour freedom has a positive significant effect on female opportunity entrepreneurship*

H4c *Higher labour freedom has no statistical significance on the necessity entrepreneurship*

H4d *Higher labour freedom has a positive significant effect on the relative presence female opportunity entrepreneurship compared with male opportunity entrepreneurship.*

### 3.5 Investment freedom

Investment freedom measures the levels at which individuals are free to decide the flow of their investment capital. This could be internally within the borders of the country or externally across the country's borders. There are varied degrees of investment restrictions. Some countries have restrictions on payments, transfers, restrictions capital transactions, and restrictions on access to foreign investment.

To ensure that entrepreneurial activities are able to generate the needed economic development, governments are promoting investment freedom to encourage entrepreneurship (Gwartney et al., 2009). Investment freedom will free idle capital into entrepreneurship, which will lead to jobs creation. Investment freedom will limit the entry and exit barriers by so doing transaction costs and regulations will be reduced for the entrepreneurs (Bennett and Nikolaev, 2019). According to Gohmann (2012), individuals who live in countries where there is much investment freedom are more likely to engage in entrepreneurship activities (P.Todaro and Smith, 2015). With opportunity entrepreneurship, which comes because of the prospects in the prevailing environment, we argue that investment freedom will promote the activities of opportunity entrepreneurship. Based on this assumption, we, therefore, proposed the following hypotheses:

H5a *Higher investment freedom has a positive significant effect on opportunity entrepreneurship*

H5b *Higher investment freedom has a positive significant effect on female opportunity entrepreneurship*

H5c *Higher investment freedom has no significant effect on the necessity entrepreneurship*

H5d *Higher investment freedom has a positive significant effect on the relative presence female opportunity entrepreneurship compared with male opportunity entrepreneurship.*

### 3.6 Financial freedom

The freedom of financial sector is the independence of banking sector from government interferences. In a situation where banking and financial sector of the economy have minimum state interference, there is smooth flow of funds. Allocation of credit is based on the market conditions. It affords the financial institutions to allocate funds to individuals and companies in timely manner. Financial freedom promotes the free flow of liquidity as banks are free to extend credit, accept deposits and conduct business in foreign currencies.

Raising capital to finance a business is a headache most entrepreneurs go through. Most entrepreneurs have to rely on banks and other financial institutions for funding because of lack of personal funds (Blanchflower and Oswald, 1998). According to the Schwab and Sala-i-Martín (2016) access to credit is a limiting factor for start-ups. The start of every business venture revolves around credit (Schumpeter, 1934). A country's financial freedom is highly depended how easy it is to access credit either from banks or other financial institutions (Schwab and Sala-i-Martín, 2016). Restrictions on the financial sector will limit the flow of credit to entrepreneurs, especially for those who requires huge sums for their businesses. A restricted financial sector will limit opportunity entrepreneurship, as entrepreneurs will not get the required funds for expansion of their businesses, which is the core objective of opportunity entrepreneurs (Reynolds et al., 2002; Hessels et al., 2008). We, therefore, contend that higher financial freedom will lead to an increase in opportunity entrepreneurship. Based on the above exposition we therefore hypothesize that:

H6a *Higher financial freedom has a positive significant effect on opportunity entrepreneurship*

H6b *Higher financial freedom has a positive significant effect on female opportunity entrepreneurship*

H6c *Higher financial freedom has no statistical a positive significance on necessity entrepreneurship*

H6d *Higher financial freedom has a positive significant effect on the relative presence female opportunity entrepreneurship compared with male opportunity entrepreneurship*.

## 4 Methodology

The study concentrated mainly on the BRICS economies. The BRICS economies are Brazil, Russia, India and South Africa. However, the inclusion of the countries was purely based on the availability of data for each of the variables. Data on India was not complete as it was not captured in most years in GEM (Global Entrepreneurship Monitor) data so it was excluded from the analysis. The data for analysis were obtained from World Bank Database, Index of Economic Freedom and Global Entrepreneurship Monitor (GEM). The data span is from 2009 to 2017.

### 4.1 Dependent variables

The study considered six separate dependent variables in the analyses. They include TEA- opportunity entrepreneurship (Total Entrepreneurship Activity), TEA-male opportunity entrepreneurship, TEA-women opportunity entrepreneurship, TEA-necessity entrepreneurship, TEA-male necessity entrepreneurship, and TEA-women necessity entrepreneurship. These variables were obtained from Global entrepreneurship Monitor database. These measurements are mainly on adult population age between 18-64 who have engaged in entrepreneurial activity in the past 24 months. This is panel data and span from 2009-2017. The opportunity entrepreneurship concerns individuals who got involved in entrepreneurship because of the opportunities the environment presents. While necessity entrepreneurship has to do with individuals who got involved in entrepreneurship as a means of survival. As an extension on previous studies, the current study looks at the gender dimension of each type of entrepreneurship.

### 4.2 Independent variables

Independent variables were obtained from the World Bank Database and Index of Economic Freedom. The annual GDP growth, population growth, and unemployment were obtained from World Bank Database. The data on property rights, financial freedom, investment freedom, labour freedom, fiscal freedom and business freedom were obtained from Index of Economic Freedom. In order to capture the impact of economic variables on entrepreneurship, we included in the analyses GDP growth, unemployment and population. GDP growth is a proxy for the county's level of growth. According to Bowen and De Clercq (2008) a growth in GDP has a positive effect on entrepreneurship. High unemployment is assumed to be a disincentive to the creation of new enterprises, as there are limited opportunities in times of crisis. Unemployment is expected to have a positive relationship with necessity entrepreneurship as people in moment of crisis will have to rely on necessity entrepreneurship as a means of survival (Verheul et al., 2002; Spencer and Gómez, 2004). It is also assumed that population growth will influence entrepreneurship positively as it will lead to emergence of new consumers creating opportunity for entrepreneurial activities. It is expected to influence both type of entrepreneurships but greater effect is expected on opportunity entrepreneurship (Wennekers et al., 2005; Autio and Acs, 2010).

In this study, it is assumed that females and males will react differently to entrepreneurial activities, hence the decision to look at the gender components of each type of entrepreneurship.

After providing a descriptive analysis of the selected variables that are used in the analysis, we proceeded to estimate some regression models of TEA*i* as a function of institutional and economic factors. The OLS model is specified as:

(1) $$TEA_{i}=\;\alpha +\sum X_{i}\beta_{i}+\mu_{i}$$

Where $TEA_{i}$∗ is the dependent variable— total entrepreneurship activity for each entrepreneurship type *i*.

$X_{i}$ is vector of institutional and economic variables influencing entrepreneurship.

α and β represent constant parameters to be estimated in the model.

$\mu_{i}\;$ represents the error term, following a standard normal distribution.

## 5 Results (see Table 1)

The Table 2 below presents the result on correlation between the variables. There exists a very high correlation between the variables, which is an indication of multicollinearity. As a result, VIF was calculated, which were above the threshold level of 10. To avoid this problem, each variable was entered into the analysis separately as recommended and used by (Fuentelsaz et al., 2015; Klapper et al., 2006). Multicollinearity has the potential to affect the precision of results (Gujarati, 2004).

**Table 1:** Descriptive statistics.

Variable	Obs	Mean	Std. Dev.	Min	Max
TEA-Opportunity	27	7.426667	3.515251	2.52	13.64
TEA-Necessity	27	4.122593	2.40127	1.12	9.74
TEA-Men Opportunity	27	8.472963	3.778977	2.87	14.56
TEA-Female Opportunity	27	6.40037	3.372972	1.97	13.02
TEA-Men Necessity	27	4.167778	2.332119	1.08	10.66
TEA-Female Necessity	27	4.077037	2.651705	1.05	11.02
GDP Growth	28	3.217801	4.236255	-7.79999	10.63614
Population Growth	28	0.762502	0.528618	0.030114	1.605752
Unemployment	28	10.73643	8.251892	4.5	25.156
Property Rights	28	36.07143	14.29563	20	50
Fiscal Freedom	28	72.96786	6.444467	65.8	86.9
Business Freedom	28	60	10.67784	46.4	76.3
Labour Freedom	28	57.98929	3.718954	49.8	63.5
Investment Freedom	28	37.32143	12.20824	20	55
Financial Freedom	28	45.35714	13.4666	30	60

**Table 2:** Correlation.

	1	2	3	4	5	6	7	8	9	10	11	12	13	14	15
TEA-Opportunity	1														
TEA-Necessity	0.8513	1													
TEA-Men Opportunity	0.9838	0.8658	1												
TEA-Female Opportunity	0.9798	0.7994	0.9282	1											
TEA-Men Necessity	0.7659	0.9562	0.767	0.7309	1										
TEA-Female Necessity	0.8637	0.9672	0.8886	0.8008	8505	1									
GDP Growth	0.3433	0.4102	0.3433	0.3242	0.5424	0.2668	1								
Population Growth	0.1362	-0.0086	0.1605	0.1059	-0.022	0.0032	-0.1711	1							
Unemployment	-0.3289	-0.3713	-0.2893	-0.3582	-0.3477	-0.3653	-0.3346	0.8648	1						
Property Rights	0.0601	-0.0965	0.0528	0.0682	-0.1859	-0.011	-0.3905	0.5643	0.4467	1					
Fiscal Freedom	-0.4177	-0.4294	-0.4359	-0.3782	-0.4496	-0.3823	-0.1238	-0.5346	-0.2854	-0.5027	1				
Business Freedom	-0.3356	-0.3934	-0.3038	-0.3576	-0.3949	-0.3645	-0.2582	0.5133	0.6155	0.5041	0.0943	1			
Labour Freedom	-0.3708	-0.2762	-0.3827	-0.3465	-0.1463	-0.3683	0.0457	-0.1171	0.0603	-0.2717	0.0771	-0.1685	1		
Investment Freedom	0.1349	-0.0139	0.1298	0.1368	-0.1136	0.0746	-0.4158	0.5635	0.4161	0.9599	-0.5993	0.3968	-0.2597	1	
Financial Freedom	0.0816	-0.1548	0.086	0.0793	-0.2818	-0.0349	-0.5269	0.8007	0.7189	0.6239	-0.2111	0.4399	-0.183	0.6026	1

The Table 3 below presents the result on opportunity entrepreneurship. Six separate models were analysed under this category. The least R-square for all the models in Table 3 is 81% an indication that at least 81% of the variations in the models are explained by the variables captured. To our surprise all, the formal institution factors were not statistically significant. With the exception of financial freedom, which has a positive sign the rest have negative coefficients in relation to opportunity entrepreneurship. This finding is completely different from the findings from extant literature about the role of formal institutions in relation to opportunity entrepreneurship (Fuentelsaz et al., 2015). The signs and non-significance contradicts what some researchers have revealed about their relationship in relation to opportunity entrepreneurship it however confirms Index of Economic Freedom Report (Gwartney et al., 2009; P.Todaro and Smith, 2015), which indicated that “the BRICS economies” (Brazil, Russia, India, China and South Africa) have shown little or no progress in economic freedom (Miller et al., 2013). Less or no economic freedom sends negative signals to entrepreneurs who have growth aspiration or mindset and deters them from engaging in opportunity entrepreneurship. Lack of economic freedom leads to crowding out of opportunity entrepreneurship, as there are no motivations for individuals to pursue their aspirations (P.Todaro and Smith, 2015).

**Table 3:** Opportunity entrepreneurship.

	1	2	3	4	5	6	7
Property Rights		-0.03433					
		(0.019925)					
Fiscal Freedom			-0.02794				
			(0.079297)				
Business Freedom				-0.05464			
				(0.033749)			
Labour Freedom					-0.08115		
					(0.087999)		
Investment Freedom						-0.03024	
						(0.025)	
Financial Freedom							0.04847
							(0.04420)
GDP Growth	0.036306	-0.00926	0.032226	0.029219	0.049151	-0.00319	0.105416
	(0.108141)∗∗∗	(0.103285)	(0.107961)	(0.102777)	(0.110185)	(0.110076)	(0.129407)
Population Growth	11.00549	11.82937	10.65601	10.95082	10.56613	11.71832	9.874071
	(1.109195)∗∗∗	(0.994923)∗∗∗	(1.516323)∗∗∗	(1.070183)∗∗∗	(1.161242)∗∗∗	(0.995868)∗∗∗	(1.317301)∗∗∗
Unemployment	-0.73294	-0.75941	-0.72031	-0.6893	-0.7046	-0.76005	-0.71549
	(0.06622)∗∗∗	(0.065104)∗∗∗	(0.078283)∗∗∗	(0.071595)∗∗∗	(0.063721)∗∗∗	(0.065537)∗∗∗	(0.063953)∗∗∗
Constant	6.687772	7.746209	8.862482	9.523454	11.38131	7.703218	4.921129
	(0.792334)∗∗∗	(1.124995)∗∗∗	(6.212782)	(1.873831)∗∗∗	(5.304026)∗∗	(1.367868)∗∗∗	(2.03353)∗∗
**R-squared**	**0.8116**	**0.8221**	**0.8129**	**0.8277**	**0.8181**	**0.817**	**0.8181**

Source: Authors' Own Calculation. ∗∗∗ at 1%, ∗∗ at 5% and ∗ at 10% sig. level.

Population growth and unemployment have statistically significant effect. The result shows that population has a positive effect on opportunity entrepreneurship confirming extant literature (Wennekers et al., 2005; Autio and Acs, 2010). Population growth leads to emergence of new markets and creates opportunity for investment. The result for unemployment shows that increase in unemployment has negative relationship with opportunity entrepreneurship (Verheul et al., 2002; Spencer and Gómez, 2004). Unemployment creates crisis and limits the opportunity for investment even as those with capital will be afraid to commit it into entrepreneurship, as there will be limited market for their produce due to limited purchasing power. Individuals who engage in opportunity have aspiration for growth, and since high unemployment will limit their potential for growth due limited market opportunity entrepreneurship will be badly affected.

The Table 4 shows the result on necessity entrepreneurship. The least R-square for all the models in Table 3 is 55% an indication that at least 55% of the variations in the models are explained by the variables captured. Six separate models were analysed under this category. Fiscal freedom and business freedom were found to be statistically significant but negatively associated with necessity entrepreneurship. The remaining formal institutional factors were not significant but have negative relationship with necessity entrepreneurship. This outcome corroborates some studies, which argued that fiscal freedom and business freedom and by extension institutional factors would not be of much benefit to necessity entrepreneurs as they do not go into entrepreneurship with growth mindset and will not take advantage fiscal freedom and business freedom to promote their activities (Reynolds et al., 2003; Hessels et al., 2008; Estrin et al., 2013; Levie and Autio, 2011). The results on property rights, labour freedom, investment freedom, and financial freedom support the hypotheses H_1C_, H_4C_, H_5C_ and H_6C_ that they have no significant effects on necessity entrepreneurship. The non-significance and negative relationship with necessity confirms the assertion that formal institutions have no effect on necessity entrepreneurship (Fuentelsaz et al., 2015).

**Table 4:** TEA-necessity entrepreneurship.

	1	2	3	4	5	6	7
Property Rights		-0.02993					
		(0.021887)					
Fiscal Freedom			-0.11282				
			(0.053676)∗∗				
Business Freedom				-0.05022			
				(0.026157)∗			
Labour Freedom					-0.05051		
					(0.096809)		
Investment Freedom						-0.01972	
						(0.024421)	
Financial Freedom							-0.04495
							(0.037673)
GDP Growth	0.962522	0.056525	0.079773	0.089738	0.104248	0.070499	0.032165
	(0.131806)	(0.124394)	(0.124965)	(0.127712)	(0.127281)	(0.136456)	(0.119269)
Population Growth	5.297166	6.015407	3.885829	5.246917	5.023662	5.761945	6.346365
	(1.200185)∗∗∗	(1.251188)∗∗∗	(1.207035)∗∗∗	(1.187801)∗∗∗	(1.119605)∗∗∗	(1.323834)∗∗∗	(1.067652)∗∗∗
Unemployment	-0.37977	-0.40284	-0.32875	-0.33966	-0.36212	-0.39744	-0.39595
	(0.086596)∗∗∗	(0.089862)∗∗∗	(0.077812)∗∗∗	(0.081413)∗∗∗	(0.079078)∗∗∗	(0.092152)∗∗∗	(0.081745)∗∗∗
Constant	3.792237	4.714961	12.57447	6.398602	6.713984	4.454327	5.430493
	(0.748803)∗∗∗	(1.060438)∗∗∗	(4.608628)∗∗	(1.79054)∗∗∗	(5.958222)	(1.226035)∗∗∗	(1.570223)∗∗∗
**R-Squared**	**0.5488**	**0.566**	**0.5953**	**0.5779**	**0.5542**	**0.5537**	**0.5608**

Source: Authors' Own Calculation. ∗∗∗ at 1%, ∗∗ at 5% and ∗ at 10% sig. level.

The result shows that unemployment has a negative significant effect on necessity entrepreneurship this implies that the motivation to engage in necessity entrepreneurship decreases with a rise in unemployment. This finding is in sharp contrast to the notion behind necessity entrepreneurship, as one would have expected unemployment to push more people into necessity entrepreneurship.

The Tables 5 and 6 below present the results on TEA-male opportunity entrepreneurship and TEA-female opportunity entrepreneurship respectively. The R-square values for Tables 5 and6 are at least 76% and 81%, respectively, an indication that at least 76% and 81% of the variations in the models of respective tables are explained. The result for TEA-male opportunity entrepreneurship shows that formal institutional factors did not play any significant role in male opportunity entrepreneurship. With the exception of financial freedom, which has a positive sign the remaining factors have negative signs. This finding is in sharp contrast to the study by (Fuentelsaz et al., 2015), which found formal institutions to have significant effect on opportunity entrepreneurship. This result confirms the Index of Economic Freedom Report, which indicates that “the BRICS economies” (Brazil, Russia, India, China and South Africa) have shown little or no progress in economic freedom (Miller et al., 2013; P.Todaro and Smith, 2015). They are economically “mostly unfree.” This implies that lack of economic freedom is an impediment to entrepreneurial activities and its development. However, there are no difference in the effect of formal institutions on both genders, thus female opportunity entrepreneurship and male opportunity entrepreneurship. However, population growth and unemployment have significant on female opportunity entrepreneurship and male opportunity entrepreneurship. Population growth has positive significant effect on both an indication that increase in population leads to discovery of new market opportunities for both males and females. Their respective effects are almost the same as they almost have the same coefficients level. The result shows that an increase in unemployment will result in a decrease in male and female opportunity entrepreneurship. Unemployment affects the two groups negatively and almost equally as their respective coefficients are almost the same.

**Table 5:** TEA- men opportunity entrepreneurship.

	1	2	3	4	5	6	7
Property Rights		-0.04223					
		(0.025555)					
Fiscal Freedom			-0.04777				
			(0.088293)				
Business Freedom				-0.05427			
				(0.042325)			
Labour Freedom					-0.11526		
					(0.118351)		
Investment Freedom						-0.03594	
						(0.030869)	
Financial Freedom							0.03887
							(0.053923)
GDP Growth	0.058598	0.00254	0.051621	0.051559	0.076841	0.011657	0.114018
	(0.141057)	(0.140144)	(0.143532)	(0.138639)	(0.136493)	(0.147642)	(0.169815)
Population Growth	11.47569	12.48916	10.87816	11.42139	10.85163	12.32284	10.56837
	(1.574481)∗∗∗	(1.443068)∗∗∗	(1.777663)∗∗∗	(1.561623)∗∗∗	(1.637636)∗∗∗	(1.482448)∗∗∗	(2.012596)∗∗∗
Unemployment	-0.74735	-0.7799	-0.72575	-0.704	-0.70709	-0.77957	-0.73335
	(0.1054)∗∗∗	(0.102753)∗∗∗	(0.107157)∗∗∗	(0.104044)∗∗∗	(0.104403)∗∗∗	(0.104623)∗∗∗	(0.108179)∗∗∗
Constant	7.44674	8.748746	11.16493	10.2632	14.11326	8.653531	6.030021
	(1.020136)∗∗∗	(1.328138)∗∗∗	(7.186561)	(2.508989)∗∗∗	(7.05424)∗	(1.610297)∗∗∗	(2.486432)∗∗
**R-Squared**	**0.7563**	**0.7701**	**0.7597**	**0.77**	**0.7677**	**0.763**	**0.7599**

Source: Authors' Own Calculation. ∗∗∗ at 1%, ∗∗ at 5% and ∗ at 10% sig. level.

**Table 6:** TEA-female opportunity entrepreneurship.

	1	2	3	4	5	6	7
Property Rights		-0.0267					
		(0.019962)					
Fiscal Freedom			-0.00484				
			(0.074827)				
Business Freedom				-0.05547			
				(0.03129)∗			
Labour Freedom					-0.0494		
					(0.082143)		
Investment Freedom						-0.0255	
						(0.024241)	
Financial Freedom							0.060418
							(0.041354)
GDP Growth	0.009133	-0.02631	0.008426	0.001938	0.016952	-0.02417	(0.095276)
	(0.095577)	(0.088611)	(0.0944)	(0.089568)	(0.104582)	(0.095488)	0.111775
Population Growth	10.52875	11.16944	10.46825	10.47325	10.26127	11.12983	9.118446
	(0.904385)∗∗∗	(0.881108)∗∗∗	(1.37359)∗∗∗	(0.846803)∗∗∗	(1.048087)∗∗∗	(0.871013)∗∗∗	(1.035256)∗∗∗
Unemployment	-0.7177	-0.73828	-0.71552	-0.6734	-0.70045	-0.74057	-0.69595
	(0.046505)∗∗∗	(0.049)∗∗∗	(0.063568)∗∗∗	(0.060644)∗∗∗	(0.051529)∗∗∗	(0.049072)∗∗∗	(0.041423)∗∗∗
Constant	5.961543	6.784639	6.337988	8.840278	8.818855	6.817808	3.759453
	(0.644643)∗∗∗	(1.05506)∗∗∗	(5.707694)	(1.627008)∗∗∗	(4.918253)∗	(1.271706)∗∗∗	(1.839861)∗
**R-Squared**	**0.8137**	**0.8206**	**0.8137**	**0.8317**	**0.8163**	**0.8179**	**0.8247**

Source: Authors' Own Calculation. ∗∗∗ at 1%, ∗∗ at 5% and ∗ at 10% sig. level.

The Tables 7 and 8 below present the results on the TEA-male necessity entrepreneurship and TEA-female necessity entrepreneurship respectively. With respect to the R-square, the Table 7 shows that at least 53% of the variations are explained by the variables. The Table 8 shows that 54% of the variations in the model are explained. The result for the TEA-male necessity entrepreneurship shows that fiscal freedom, business freedom and financial freedom have significant negative effect on male necessity entrepreneurship. This result supports literature, which indicates that business freedom presents many opportunities to job seekers and limits the chances of individuals going into necessity entrepreneurship as a means of survival (Reynolds et al., 2002; Hessels et al., 2008; Fuentelsaz et al., 2015).

**Table 7:** TEA- men necessity entrepreneurship.

	1	2	3	4	5	6	7
Property Rights		-0.03362					
		(0.024977)					
Fiscal Freedom			-0.13373				
			(0.047426)∗∗∗				
Business Freedom				-0.05258			
				(0.027608)∗			
Labour Freedom					0.007311		
					(0.098482)		
Investment Freedom						-0.02395	
						(0.030046)	
Financial Freedom							-0.08961
							(0.037311)∗∗
GDP Growth	0.198237	0.153612	0.178703	0.191416	0.197079	0.166965	0.070465
	(0.121602)	(0.114219)	(0.110331)	(0.115688)	(0.124414)	(0.127647)	(0.106453)
Population Growth	4.159932	4.966708	2.487039	4.107318	4.199514	4.724297	6.251749
	(1.211855)∗∗∗	(1.364195)∗∗∗	(1.163531)∗∗	(1.221928)∗∗∗	(1.220118)∗∗∗	(1.46525)∗∗∗	(0.896767)∗∗∗
Unemployment	-0.29097	-0.31689	-0.2305	-0.24898	-0.29353	-0.31244	-0.32323
	(0.081554)∗∗∗	(0.087885)∗∗∗	(0.069552)∗∗∗	(0.082772)∗∗∗	(0.079848)∗∗∗	(0.091451)∗∗∗	(0.066173)∗∗∗
Constant	3.409247	4.445712	13.81905	6.138246	2.986407	4.213201	6.675485
	(0.695891)∗∗∗	(1.103326)∗∗∗	(4.061674)∗∗∗	(1.782294)∗∗∗	(5.914965)	(1.340989)∗∗∗	(1.616677)∗∗∗
**R-Squared**	**0.5338**	**0.5568**	**0.6031**	**0.5677**	**0.5339**	**0.5416**	**0.5844**

Source: Authors' Own Calculation. ∗∗∗ at 1%, ∗∗ at 5% and ∗ at 10% sig. level.

**Table 8:** TEA-female necessity entrepreneurship.

	1	2	3	4	5	6	7
Property Rights		-0.02562					
		(0.022728)					
Fiscal Freedom			-0.09303				
			(0.064012)				
Business Freedom				-0.04794			
				(0.029232)			
Labour Freedom					-0.10719		
					(0.105174)		
Investment Freedom						-0.01469	
						(0.023772)	
Financial Freedom							-0.00129
							(0.043186)
GDP Growth	-0.00392	-0.03793	-0.01751	-0.01014	0.013048	-0.02311	-0.00576
	(0.15023)	(0.144287)	(0.148078)	(0.14843)	(0.137192)	(0.155176)	(0.138272)
Population Growth	6.390215	7.005032	5.22646	6.342247	5.809864	6.736533	6.420433
	(1.366878) ∗∗∗	(1.369431) ∗∗∗	(1.357528) ∗∗∗	(1.34411) ∗∗∗	(1.182546) ∗∗∗	(1.426318) ∗∗∗	(1.389666)∗∗∗
Unemployment	-0.46538	-0.48513	-0.42331	-0.42709	-0.42795	-0.47855	-0.46585
	(0.103604) ∗∗∗	(0.105821) ∗∗∗	(0.09556) ∗∗∗	(0.093973) ∗∗∗	(0.089783) ∗∗∗	(0.107547) ∗∗∗	(0.102858)∗∗∗
Constant	4.168572	4.958426	11.41019	6.656618	10.36824	4.661911	4.215754
	(0.87481) ∗∗∗	(1.174839) ∗∗∗	(5.48569)∗∗	(2.05104) ∗∗∗	(6.555923)	(1.304963)∗∗∗	(1.733552)∗∗
**R-Squared**	**0.5374**	**0.5478**	**0.5634**	**0.5592**	**0.5575**	**0.5397**	**0.5374**

Source: Authors' Own Calculatio. ∗∗∗ at 1%, ∗∗ at 5% and ∗ at 10% sig. level.

However, the result on TEA-female necessity entrepreneurship shows that no formal institutional factors play significant role in the female necessity entrepreneurship. This finding supports hypotheses H_1C_, H_2B_, H_3C_, H_4C_, H_5C_ and H_6c_. The results corroborates the research findings by (Fuentelsaz et al., 2015). For male necessity entrepreneurship, fiscal freedom, business freedom and financial freedom affect it negatively and significant at 1%, 10% and 5%, respectively.

The result on the population growth and unemployment are significant for both female necessity entrepreneurs and male necessity entrepreneurs. Whiles population growth affect them positively unemployment negatively affects them. Its effect on female necessity entrepreneurs is however greater compared to male necessity entrepreneurs as the coefficients of the female necessity entrepreneurs are greater than male necessity entrepreneurs for all the six models. This shows that females are more responsive to necessity entrepreneurship with population change than males. The result on unemployment shows that male necessity entrepreneurship and female necessity entrepreneurship are negatively affected when unemployment increases. This implies that the motivation to engage in necessity entrepreneurship decreases with a rise in unemployment. This finding is in sharp contrast to the notion behind necessity entrepreneurship, as one would have expected unemployment to push more people into necessity entrepreneurship. The result however shows that the effect of unemployment is much greater on female necessity entrepreneurship compared to male necessity entrepreneurship.

## 6 Discussion

The main objective of the study is to assess whether formal institutions have influenced opportunity entrepreneurship and necessity entrepreneurship. The gender component of each of the entrepreneurship was also looked at. The result shows that formal institutions in BRICS economics have not contributed significantly positive to the development of entrepreneurship. With the exception of financial freedom with a positive sign even though not statistically significant, the remaining formal institutions factors have negative signs and statistically insignificant for the opportunity entrepreneurship. With the exception of fiscal freedom and business freedom, which were negatively statistically significant at 5% and 10%, respectively, for necessity entrepreneurship the remaining variables were not statistically significant. This goes to support the long held notion that formal institutions do no play significant role in necessity entrepreneurship (Fuentelsaz et al., 2015). These findings for the opportunity entrepreneurship are in sharp contrast to early researches by (Fuentelsaz et al., 2015; Bowen and De Clercq, 2008; Levie and Autio, 2011) who found a positive relationship between formal institutions and opportunity entrepreneurship. The signs however corroborates research findings by (McMullen et al., 2008; Valdez and Richardson, 2013), which indicated that formal institutions hampers the development of opportunity entrepreneurship. For the economic variables, the results shows that population growth leads to opportunity entrepreneurship and necessity entrepreneurship development. This corroborates the study by Fuentelsaz et al. (2015), which revealed that population growth leads to emergence of new markets and also forces individuals to engage in necessity entrepreneurship as there will be many people competing for the few available opportunities (Wennekers et al., 2005; Autio and Acs, 2010; Fuentelsaz et al., 2015). The magnitude of the effect varies sharply across types of entrepreneurship but with greater impact on the opportunity entrepreneurships. The result shows that unemployment has a negative effect on both opportunity entrepreneurship and necessity entrepreneurship. The result support extant literature on the relationship between unemployment and opportunity entrepreneurship (Spencer and Gómez, 2004; Verheul et al., 2002; Fuentelsaz et al., 2015) it however contradicts results on its relationship with the necessity entrepreneurship.

The result on TEA-male opportunity entrepreneurship and TEA-female opportunity entrepreneurship reveal another interesting finding. With the exception of business freedom, which shows a negative significant effect on TEA-female opportunity entrepreneurship, the remaining formal institutional factors have no significant effect on TEA-male opportunity entrepreneurship and TEA-female opportunity entrepreneurship. The negative significant effect of business freedom on female opportunity entrepreneurship corroborates Index of Economic Freedom Report (Gwartney et al., 2009; Miller et al., 2013), which indicated that there is no economic freedom in BRICS economies “economically mostly unfree.” It implies that TEA-female opportunity entrepreneurs reacts are more to restrictions on business freedom compared to male-opportunity entrepreneurs. This finding corroborates the studies by (Spencer and Gómez, 2004; Levie and Autio, 2011), which indicated that restrictions on business freedom demotivates and decreases entrepreneurship, especially entrepreneurs with growth mindset. The study by Klapper et al. (2006), (P.Todaro and Smith, 2015) also indicated that administrative regulations deter people from creation of new ventures especially opportunity entrepreneurs. The finding further corroborates the study by (Bilgin et al., 2017), which indicated that countries with less quality institutions are barriers to trade as players such as employers, employees and stakeholders are discouraged from embarking on entrepreneurial activities. The BRICS economies have recently started moving away from market led economies, as there is an increased in resources nationalization, protectionism and a lack of momentum for additional market-oriented reforms to increase the size of private sector. The study, however, refutes earlier finding by Fuentelsaz et al. (2015) who found a positive relationship between business freedom and opportunity entrepreneurship even though their study did not consider the gender components of opportunity and necessity entrepreneurship. According to (McMullen et al., 2008; Estrin et al., 2013) lack of fiscal freedom affects entrepreneurship negatively as it siphons capital from private sector to the public sector thereby impeding entrepreneurial activities.

The result for the TEA-male necessity entrepreneurship shows that fiscal freedom, business freedom ad financial freedom have negative effect on TEA-male necessity entrepreneurship. This implies that the lack of fiscal freedom, business freedom and financial freedom will lead to a decrease in male-necessity entrepreneurship. This finding contradicts the study by Amin and Haidar (2012) who argued that cumbersome business procedures rather promotes informal sector (necessity entrepreneurship) as they often do not register their operations as way avoiding complex procedures.

On the financial freedom, the result shows a negative significant relationship with TEA-male necessity entrepreneurship. This finding contradicts the studies by (Hessels et al., 2008; Reynolds et al., 2003), which indicated that restrictions on the financial freedom affects only entrepreneurs with growth mindset (opportunity entrepreneurs) and not necessity entrepreneurship. Financial restrictions limit the amount of capital available for entrepreneurial activities. The results show that restricted business freedom negatively affects TEA-male necessity entrepreneurship. This finding contradicts the study by Amin and Haidar (2012) who argued that cumbersome business procedures rather promotes informal sector (necessity entrepreneurship) as they often do not register their operations as way avoiding complex procedures.

## 7 Conclusion

In this paper, we look at how formal institutions influence entrepreneurship in BRICS economies using OLS. The datasets are obtained from Global Entrepreneurship Monitor (GEM) World Bank Database, and Index of Economic Freedom. Our research focuses on a more fine-grained analysis comparing opportunity entrepreneurship, which is far more linked to economic growth, with necessity entrepreneurship, and which usually arises because of the lack of labor alternatives. The study also looks at how changes in some key economic indicators have influenced various types of entrepreneurship. We observe that population has a positive impact on the development of all categories of entrepreneurship. Population has a higher positive impact on opportunity entrepreneurship, male-opportunity entrepreneurship and female-opportunity entrepreneurship compared to necessity entrepreneurship, male-necessity entrepreneurship and female-necessity entrepreneurship. In terms of gender, population growth has a higher positive impact on male-opportunity entrepreneurship compared to female-opportunity entrepreneurship. Under necessity entrepreneurship, it shows that population growth has more positive impact on female-necessity entrepreneurship than male-necessity entrepreneurship. It also emerges that unemployment has a negative impact of all categories of entrepreneurship. The magnitude, however, vary sharply across different type of entrepreneurship. Unemployment has a greatest negative impact on opportunity entrepreneurship, male opportunity entrepreneurship and female opportunity entrepreneurship. Even though, unemployment has negative impact on the necessity entrepreneurship, male-necessity entrepreneurship and female-necessity entrepreneurship, the magnitude of the impact is less compared to opportunity entrepreneurship category. We observe that fiscal freedom and business freedom have negative impact on necessity entrepreneurship. Business freedom has a negative effect on the female-opportunity entrepreneurship. It emerges that fiscal freedom and business freedom have a negative impact on the male-necessity entrepreneurship.

The study extends the discussions on the topic by looking at the gender component of each type of entrepreneurship. Though results on the formal institutions are not statistically significant, the study shows that formal institutions in the BRICS economics have negative association with entrepreneurship with exception of financial freedom, which has a positive association with opportunity entrpreneurship, female-opportunity entrepreneurship and male-opportunity entrepreneurship. It emerged that population growth has contributed positively to all types of entrepreneurship. Unemployment has a negative effect on all types of entrepreneurship.

For public policy, the study recommends for removal of bottlenecks associated with the functioning of formal institutions to realize their full benefits to the development of opportunity entrepreneurship so to propel the growth of various economies.

Our study has the following limitations: the opportunity-necessity dichotomy can be interpreted differently depending on the country. The methodology used by GEM tries to be uniform in the regions where the study is conducted, but the concept of opportunity may differ from country to country. Second, this dichotomy could be slightly restrictive by ignoring further classifications of entrepreneurship such as commercial versus social entrepreneurship, or formal versus informal entrepreneurship.

In addition, the study looks at BRICS economies, which institutions may vary sharply at country specific level as some may have well functioning formal institutions than others. These are limitation of the study. For future research, the study recommends for a country specific and further classification of entrepreneurship analyses on the effect of formal institutions on entrepreneurship.

## Declarations

### Author contribution statement

T.B. Udimal, M. Luo and E Liu: Conceived and designed the experiments; Analyzed and interpreted the data; Wrote the paper.

N.O. Menash: Analyzed and interpreted the data; Wrote the paper.

### Funding statement

This research did not receive any specific grant from funding agencies in the public, commercial, or not-for-profit sectors.

### Competing interest statement

The authors declare no conflict of interest.

### Additional information

No additional information is available for this paper.
